# A Review of the Model/Rival (M/R) Technique for Training Interspecies Communication and Its Use in Behavioral Research

**DOI:** 10.3390/ani11092479

**Published:** 2021-08-24

**Authors:** Irene M. Pepperberg

**Affiliations:** 1 Department of Psychology, Harvard University, Cambridge, MA 02138, USA; impepper@media.mit.edu; 2 The Alex Foundation, 30 Curry Circle, Swampscott, MA 01907, USA

**Keywords:** animal–human communication, interspecies communication, vocal learning, animal cognition, model/rival training, symbolic reference, grey parrots

## Abstract

**Simple Summary:**

Comparing the cognitive capacities of nonhumans to those of humans can be quite difficult, particularly given that humans can be questioned directly (e.g., “How many?”, “What color?”) but that most nonhumans must be tested by various indirect means that might not demonstrate the full range of their capacities. A few nonhumans, however, have acquired some level of symbolic representation (e.g., labels for items such as physical objects and their attributes, for concepts and relations among these items and concepts, and for actions that can be carried out on or with these items), which allows for a limited form of interspecies communication that can be used for direct questioning. Why have so few nonhumans acquired this skill, and what are the advantages of having it? I describe a specific training procedure, the Model/Rival (M/R) protocol, that enabled several Grey parrots to learn some level of referential communication; I discuss the specific elements of such training that are both necessary and sufficient for successful acquisition and how lack of any of these elements can cause failure. I also describe some experiments that were facilitated by interspecies communication, and how acquisition of this ability might affect the extent to which nonhumans can process information.

**Abstract:**

In this paper, I will review the Model/Rival (M/R) technique that has been used to establish interspecies communication with Grey parrots (*Psittacus erithacus*). I will describe the original format developed by Todt, the relationship to other forms of observational learning outlined by other researchers, and the adaptations that I devised. I will describe how my undergraduate trainers and I isolated the various components that constitute the technique and explain how each is necessary, but how only the combination of *all* components is sufficient for successful implementation—and how improper implementation can lead to failure. I will briefly summarize the results of proper implementation—including the importance of interspecies communication itself as a technique for studying animal cognition.

## 1. Introduction

Determining the cognitive capacities of nonhumans is not a simple task; just the act of devoting a special issue to the topic makes the problem abundantly clear. A form of the Heisenberg uncertainty principle always exists when performing such studies—i.e., the process by which measuring occurs can affect the result. Moreover, given that most such experiments are comparative in nature and that human cognition is the standard against which nonhuman abilities are most often measured, the fact that humans and nonhumans are generally tested via very different techniques complicates the issue even further. For example: other than in studies with pre-verbal infants, humans are most often tested using their language abilities—by simply being asked pertinent questions—whereas nonhumans are mostly incapable of being tested in that manner. However, what about those nonhumans who actually can be questioned in the same manner as humans? Specifically, what about nonhumans that have acquired some level of symbolic representation (a term I use interchangeably with symbolic reference): that is, what about nonhumans who understand the semantic and pragmatic use of noniconic symbols—be they auditory/vocal, manual, or lexical—to stand for (but not be limited to) items such as physical objects and their attributes, various concepts, relations among these items and concepts, and actions that can be done to or with these items? Here I am utilizing Deacon’s assertions about reference [1]; that reference is not present when a label or sign is simply *associated* with something (e.g., as is a green button that, when pressed in a specific situation, delivers various food items to relieve hunger, whereas a red one does not), but is present if the label actually *represents* something independent of context (e.g., “green” describes the color of grass, a certain wavelength of light, or the skin color of a famous fictional entity from a far-off planet). Thus, any speaker of English would instantly understand what is being communicated by the label “green” in the latter case—as would a nonhuman who has also acquire some level of symbolic reference.

Such nonhumans can be tested via the use of interspecies communication [2]. Interspecies communication (a) directly states the precise content of questions to be asked—animals need not determine the nature of a question through hundreds (if not thousands) of instances of trial-and-error learning, thus making the task efficient; (b) incorporates research showing that social animals may respond more readily and accurately within an ecologically valid social context; (c) allows straightforward data comparisons among species, including humans; (d) is an open, arbitrary, creative code with enormous signal variety, enabling an animal to respond in novel, possibly innovative ways that demonstrate greater competence than the required responses of operant paradigms, and allows researchers to examine the exact nature and extent of information an animal perceives; (e) allows rigorous testing that avoids expectation cuing: subjects can be made to choose responses from their entire repertoire rather than from a subset relevant only to a particular topic. In some instances, interspecies communication via symbolic reference may simply be a means to more facilely demonstrate nonhumans’ inherent capacities; in other instances, however, it may also significantly affect their abilities to process information [3,4,5].

Although I will spend some time discussing how acquisition of symbolic representation may actually affect cognitive capacities (see Section 7), the main portion of this paper will be devoted to reviewing exactly how at least one species, the Grey parrot (*Psittacus erithacus*), was taught such a form of interspecies communication. I will review the various components that constitute the training technique, explain how each is necessary, and demonstrate how only the combination of *all* components is sufficient for successful implementation.

## 2. Choice of Subject Species

In the late 1960s and early 1970s, several projects were initiated in order to train nonhumans to communicate with humans. Their choices of species were based on the idea that subjects should, like the great apes, have a close phylogenetic relationship to humans (reviewed in [6]) or, like the cetaceans, at least have large brains relative to their body size (reviewed in [7]); the idea was that possibly latent abilities in their own communication systems would be stimulated or that their cognitive capacities would provide the bases for training to succeed. At the time, the idea of using a bird—a creature evolutionarily far removed from humans and with a brain the size of a shelled-walnut—was considered absurd. Nevertheless, considerable data existed on the vocal communication abilities of birds in general—with respect to usage and especially for parallels between avian vocal learning and young children’s early speech acquisition—and on Grey parrots in particular (reviewed in [5]). Other papers commented upon the exceptional clarity of Grey parrots’ reproduction of human speech [8,9], and still other studies documented the intelligence of this particular species (reviewed in [2,5]). Thus, I decided to obtain a Grey parrot and begin its training in a laboratory setting [2].

## 3. The First Model/Rival Technique and Social Modeling Theory—Some History

Despite the material discussed above, until my research birds such as Grey parrots were still consistently dismissed as mindless mimics [10], and early studies attempting to establish interspecies communication with mimetic birds, having had negligible success, did little to alter that view (reviewed in detail in [11]). Such projects, using operant conditioning and nonreferential food rewards [12,13], generally confounded the label of the object or action being trained with that of the unrelated food reward, preventing the subject from making appropriate associations between the human labels being taught and their actual meaning [2,5,11]. With such training, all labels being taught simply became requests for food by a hungry subject—i.e., one that was usually kept below its free-feeding weight. Birds in these studies not only failed to learn referential speech but also failed to learn much speech at all. A subsequent study [8], primarily interested in examining antiphonal duetting in Grey parrots, instead used a modeling technique (a form of observational learning), in which one human was exclusively a cooperative partner (the trainer) of the parrot, while another human acted both as a model (M) for the bird’s responses and as a rival (R) for the attention of the trainer (hence the term M/R training). The trainer interacted with both the parrot and human acting as a model, but the model never interacted with the parrot. The vocal patterns to be learned were not meaningful, although they gave the appearance of conversation (e.g., “What is your name?” “My name is Lora”). As a consequence of this procedure, each parrot engaged in verbal interaction solely with its particular trainer; learning of verbal patterns from other trainers was inhibited, as was the ability of the bird to transfer its responses to someone other than the principal trainer. However, birds in this program did acquire novel vocalizations rapidly (generally less than one month after introduction, usually within 3–6 days). The important difference between this study and previous ones was the introduction of social interaction—a not entirely surprising addition, given that communication is a social act and that in nature birds primarily learn their communication skills from other birds [8]. 

This study caused me to delve deeper into the various issues involved in determining how to establish referential communication and led to my examining research on social modeling theory and its applications [11,14,15]. Social modeling theory was the outcome of social psychologists’ attempts to determine the underlying mechanisms of what they considered real-world learning (including observational learning), in contrast to the laboratory-based behavioristic, operant associationist paradigm common at the time [16]. Social modeling theorists proposed that “provision of models not only serves to accelerate the learning process, but also, in cases where errors are dangerous or costly, becomes an essential means of transmitting behavior patterns” [16], p. 54. In an attempt to devise procedures to enable humans to overcome strong inhibitions or phobias, they derived additional aspects of the theory; the idea was that, by determining how the toughest learning problems were resolved (e.g., showing how a subject could overcome a snake phobia by a combination of observing others handling snakes, watching someone else model the process of overcoming *their* initial hesitation about handling snakes, and then imitating the actions of this successful person), they could make some inferences about general learning mechanisms [17]. Overall, these researchers developed a theory that emphasized how attention, comprehension, and motivation affected learning. The theory thus consists of a set of principles that describe the optimal form of social input for any type of learning [14]. My understanding of the theory led me to believe that by modifying the original M/R protocol so as to incorporate aspects of a subset of four of these principles, I would succeed in enabling nonhumans—in my case, Grey parrots—to acquire some form of interspecies communication [2,11]. I describe each of these principles in turn, and then explain how I used them to revise the M/R procedure. (Note that in the following general discussion, the term “student” refers to any individual that is learning a task, whether human or nonhuman).

### 3.1. Principle 1

One principle states that the student’s level of competence must be taken into account during all aspects of training. Researchers had already determined, for example, that human children most easily and most often acquire whatever is just slightly beyond their current abilities—whether it be new labels, actions, etc. (e.g., [15,18,19,20,21]). Specifically, with respect to modeling, interactions that model a new behavior that differs only slightly from an existing behavior or that encode only slightly unfamiliar information are most easily learned [11].

A corollary of this principle is that for learning to proceed efficiently, both tutor and model must constantly adjust their demonstration so that it will adapt to—and thus continue to challenge—a student’s increasing knowledge (note [20]). Finding the appropriate level of input requires careful balancing. Input that is too simple may be ignored because the learner loses interest; input that is too advanced for the learner’s level may similarly be ignored (e.g., as being irrelevant). As trainers interact with the student, they must therefore consistently determine the student’s current level, and make upward or downward adjustments; the process is recursive and continuous, with all actors working in concert [11].

### 3.2. Principle 2

A second principle of this theory is that the modeling must help the student understand how new material relates to current problems and what advantage is conferred by learning the new material. Practically, training is therefore most effective when: (a) the student sees and then practices the targeted behavior under conditions similar to those in its regular environment, and (b) the appropriate use and consequence of the behavior are explicitly demonstrated (e.g., [14,17,22,23]). Thus, the trainers must provide some motivation for the subjects to acquire the requisite knowledge (note [20]). For example, subjects who are shown exactly how a novel utterance can be used to request a desired object are likely to learn more readily than if they were in situations without such demonstrations. This principle ties into functionality and contextual applicability, which will be discussed in detail below.

### 3.3. Principle 3

A third principle states that the effectiveness of the training is related to the intensity of the interaction between a student and the models. Here, intensity is viewed as the extent to which tutors arouse a response in a student [17] and, again, requires a careful balance between extremes [24]. Too little intensity, and the student will likely ignore the training. Moreover, overly nurturant models may inhibit learning by preventing students from experimenting or attempting to go beyond their current levels (note the tie-in to the principle concerning challenging a student intellectually). However, increasing the intensity of interaction may not always increase learning. Overly aggressive models may arouse fear or counter-aggression strong enough to block processing of any input.

### 3.4. Principle 4

The fourth principle states that the more that inhibition or resistance exists towards learning, the more important are the first three principles. The fourth principle is particularly relevant when the task involves acquiring what I call *exceptional* communication [25]—communication characterized by vocal learning that is unlikely to occur in the normal course of development, such as nonhuman acquisition of symbolic reference, particularly with respect to the likelihood of nonhuman acquisition of the more complex elements of a human system. The term “exceptional” may also imply some resistance toward acquiring the targeted behavior. Thus, for exceptional learning to occur, social modeling theory predicts that tutor/models be even more attuned to the student’s level, that interactions be even more balanced with respect to intensity, and that demonstrations be even more explicit as to the real world uses and consequences of a targeted behavior than during normal learning [2,11]. This principle was especially crucial for understanding how I adapted social modeling theory to teach a Grey parrot to communicate with humans and to use this communication to examine his cognitive abilities.

### 3.5. Adapting the Principles to Training

Social modeling theory overall provides a framework for devising optimal training procedures—i.e., deciding upon optimal forms of input—but this framework must be put into practice. To wit, what factors characterize such optimal input? From the principles described above, optimal input should (a) correlate well with specific aspects of an individual’s environment (i.e., be “referential” [26]), (b) have functional meaning relevant to the individual’s environment (also known as “contextual applicability” or utility), and (c) be socially interactive. I describe these factors in some detail elsewhere (e.g., [11,27,28]); however, they bear re-examination here.

*Reference*—reference, for the most part, involves content; that is, what signals “are about” [26]. Reference, although generally defined as the direct relationship between a signal and an object or action, is not always easily determined. Signals, for example, may have multiple (if somewhat related) cultural meanings. Thus, “parrot” generally refers to one of a set of brightly colored birds (what [26] describes as an “external referent”), but may also refer to an action, as to “parrot”, or mindlessly mimic, a behavior. Similarly, in nature an alarm call may refer to either the predator or the action that the emitter is about to take—or both. The more explicit the referent of a signal, however, the more easily the signal appears to be learned.

*Functionality*—functionality (also known as contextual applicability) involves the pragmatics of signal use: when a signal is to be used and the effects of using information in the signal [11,25]. Functionality is demonstrated by showing when using a signal is advantageous and the specific advantage gained by its use. Environmental context (e.g., the situation, intonation) also factors into the manner in which a signal functions. For example, a comment such as “My, don’t we look nice today” said with one type of intonation will have one meaning and effect for a little girl in a party dress, but a different meaning and effect when said with a different intonation to a hungover friend [11]. Functionality also helps define reference; that is, context defines “parrot” as either a noun or a verb. As with reference, the more explicit a signal’s functionality, the more readily the signal appears to be learned.

*Social interaction*—the three major functions of social interaction can be clarified by examples [11]. First, social interaction can highlight which of several environmental components should be noted; e.g., a subject can be directed to an object’s shape to learn shape labels (“Look at the blocks. The *shape* of this one is *square*; the *shape* of that one is *round*”). Next, social interaction can emphasize common attributes—and thus possible underlying rules—of diverse actions (i.e., “*Give* me the *ball*” versus “*Give* me the *block*” versus “*Take* the *ball*”). Finally, social interaction is what allows input to be continuously adjusted to match the receiver’s level (“Yes, you picked a ball in this pile of toys! Now can you find the *red* ball?”). Interaction may also provide a contextual explanation for an action and demonstrate its consequences (“There are lots of toys in the box! Tell me which one you want, and you can have it.”). Interactive input of various forms thus facilitates learning.

In sum, reference and functionality concern how a label is used in the world, social interaction highlights various components of its use, and all three are necessary for meaningful learning [11]. Importantly, reference and functionality are distinct from what associative learning theory calls “reinforcement”—reinforcement is some general, positive outcome that is associated with an action (e.g., food received for hitting a button, whether the button-press in the task involves distinguishing colors, shapes, or grids), whereas reference and functionality are specific to a particular outcome out of many possibilities (e.g., “want red apple” specifies the action, a particular type of fruit and a particular varietal). I thus reasoned that, in order to teach a parrot to communicate with humans, my training procedure needed to take these factors into account. My most important insight, however, was the hypothesis that a parrot’s acquisition of a human-based code was a form of exceptional learning. Given the earlier failures, I believed that despite these birds’ abilities to reproduce all sorts of sounds, some strong inhibition existed towards learning to use allospecific sounds in a functional manner. I further believed that, to overcome this inhibition, training would have to be carefully adjusted to the parrot’s abilities and include intense interactions and extremely clear demonstrations of reference and functionality [11]. Could I modify Todt’s [8] technique appropriately? By using a form of observational learning, he had demonstrated the effectiveness of social interaction; would I succeed if I figured out how to incorporate referentiality and functionality, and extend and improve the level of social interaction?

## 4. The Second M/R Protocol

In honor of Todt’s breakthrough, I continued to call the revised training protocol the M/R procedure. However, several modifications were made in this new design. The major changes are the demonstration of referential and contextual use of labels, and of corrective feedback among two human trainers and the parrot in order to demonstrate the targeted vocal behavior [2]. The parrot watches and listens as one trainer presents objects to, and queries the other trainer about, various items (e.g., “What’s here?”, “What color?”)—items in which the bird has already demonstrated some interest. Because the reward for uttering “x” is x and only x (an *intrinsic* reward), and not an irrelevant item like a piece of food (an *extrinsic* reward), the bird thereby observes the closest possible connection between the object and the label to be learned. The trainer gives praise and transfers the named object to the human partner to reward correct answers. Incorrect responses are punished by scolding and temporarily removing items from sight. Thus, the second human is a model for the parrot’s responses, and its rival for both the trainer’s attention and acquisition of the item. The second human also illustrates consequences of errors. The model must try again or talk more clearly if the response was deliberately incorrect or garbled; that is, the model is subject to corrective feedback, which the bird observes. The parrot is included in these interactions, being queried and rewarded for successive approximations to correct responses; training is adjusted to its performance level. If a bird is inattentive or accuracy regresses, trainers threaten to leave. Note that the procedure also clearly demonstrates how the two trainers jointly attend to the object in question, another aspect of social interaction input thought to be important for acquisition of referential labels [29].

Unlike M/R procedures used by other researchers (see [30]), ours also exchanges roles of trainer and model to emphasize the importance of three-way interactions. The parrot thus sees how questioner and respondent inhabit both roles, and how the procedure causes environmental change (i.e., the transfer of the designated item). Role reversal also counteracts an earlier methodological problem [8]. Birds whose trainers always maintained their respective roles responded only to that human questioner. After several demonstrations and role reversals, the parrot itself is questioned—by both humans in their turn as trainer—and attempts at the label are rewarded with the object. The humans will then use these attempts themselves, with the person acting as trainer responding with phrases such as “That’s close, say better!” or “Talk clearly”, and giving the model another chance to respond appropriately. With our system, birds respond to, interact with, and learn from any human and, importantly, acquire the ability to ask questions themselves [11].

Although giving the parrot the object it had just labeled emphasized the referentiality and functionality of the label, this procedure also, of course, confounded identification of an object with the request for the object. We thus subsequently had to teach the birds (again through the M/R technique) to use “I want x”, which enabled them to request unrelated, preferred rewards while learning labels for various items that they found of little or no inherent interest [31]. Specifically, the bird could identify object *y* with label “y”, receive *y*, toss it, and then request *x* as the reward.

We maintained strict controls during training and testing (discussed in [5,13]), but outside of these formal sessions, we tried to provide as much vocal and social stimulation as possible, providing human interaction to substitute for that which this single bird would have received from his flock-mates in nature. He was allowed free access (contingent upon his vocal requests; e.g., “Wanna go gym”) to all parts of the laboratory for the ~8 h/day that trainers were present; in fact, trials could occur at various locations. He was confined to a standard cage (~62 × 62 × 73 cm) only during sleeping hours. Water and a standard seed mix for psittacids (sunflower seeds, dried corn, kibble, oats, safflower, etc.) were available continuously; fresh fruits, vegetables, specialty nuts (cashews, almonds, pecans, walnuts) and toys (keys, pieces of wood, paper, rawhide, etc.) were provided at his vocal requests (e.g., “I want cork”). Once he learned how to question his trainers, he could also ask them for the labels of novel objects/colors/shapes in the laboratory (e.g., “What’s here?”, “What toy/matter/color/shape?”).

With the help of this modified technique and these housing conditions, my most successful subject, Alex, acquired labels for over 100 different objects, seven colors, five shapes, exact numbers (up to eight), and categories; he acquired concepts of same/different, bigger/smaller, addition, numerosity, something akin to ‘zero’, and functional use of phrases such as “I want x” and “Wanna go y” where x and y were appropriate object and location labels (e.g., [4,11,32,33,34]). In a subsequent section (Section 7), I will discuss some of his capacities and those of other Grey parrots. For now, I concentrate on training issues, and one issue in particular: how the initial studies did not enable us to learn exactly why the M/R technique was so successful; we could not yet specify which training elements were necessary and sufficient. Thus, additional, different types of experiments, each of which carefully eliminated specific aspects of training, were needed to determine these criteria.

## 5. Experiments That Eliminate Aspects of M/R Training

These different experiments had to wait, for the most part, for the acquisition of additional subjects. The rationale was that, had I significantly changed Alex’s training protocols, he might have ceased learning simply because I had made any changes, not because of the quality of the changes. New subjects, lacking a history of training, would not be influenced by such prior experiences. Hence, the introduction into the lab, at various times, of the (then juvenile) Grey parrots Kyo, Alo, Griffin, and Arthur. For these new subjects, I could provide input that varied with respect to various amounts of reference, functionality, and social interaction. I therefore contrasted sessions involving standard M/R tutoring on some labels with sessions on other labels that used audiotapes, videos, as well as variants of M/R and video input that involved different levels of human interaction. Moreover, to see what could happen if we did make changes in Alex’s learning environment, I also exposed him to a variant of M/R training.

### 5.1. Audiotape Instruction

To test how learning might be affected if input totally lacked reference, context, and social interaction, I used two juvenile parrots, Alo and Kyaaro, contrasting their training via audiotapes with the standard M/R protocol. In the audiotape sessions, juveniles sat on a perch and listened to tapes while isolated from each other and the rest of the laboratory, and no objects were associated with the sounds presented over the speaker [35]. The two labels used for one bird in the audiotape condition were the two labels used for the other bird with the M/R procedure, to ensure that any difference in acquisition would not be a consequence of the form of the label. Notably, all four labels had been acquired by Alex, so I knew that they could be learned by a Grey parrot [11,35]. Both Kyaaro and Alo had already demonstrated that they found the objects to which these labels referred to be of considerable interest. To ensure that sessions paralleled M/R procedures, audiotapes consisted of the audio portion of a video of one of Alex’s M/R sessions for each label. Although Alex already knew the labels being used in these videos [11], his M/R sessions were not structured as reviews, but replicated actual training. For him, however, the sessions *were* reviews and thus were not engaging, and he occasionally erred or interrupted with requests for other items and changes of location, which enabled us to demonstrate the same corrective feedback as usual [2,11]. As in regular live M/R sessions, trainers switched roles and occasionally deliberately erred. The audio was analyzed (Kay 5500 DSP Sona-Graph, Kay Electric Company, Lincoln Park, NJ, USA) to ensure that it was not degraded compared to that of Alex “live”. Alo and Kyaaro each learned the two labels trained via the M/R procedure but neither label trained via audiotape. This study clearly showed that absence of all three aspects of input hindered acquisition, but not whether the presence of some subset or some intermediate amount of these types of input would be sufficient to engender learning.

### 5.2. M/R Variant 1

What would happen if we provided plenty of social interaction (more than in Todt’s original study [8]), but eliminated reference and functionality? The experiment to test this with Alex was part of research on ordinality, counting, and serial learning [11]. We wanted to teach him a number line—that is, to recite a set of number labels in order of their value, in ways similar to the behavior of children in their early stages of numerical education [36]—but did not want this training to affect our other numerical studies. Thus, while Alex watched his trainers, one human asked a question, “Say number?” and the other uttered a string of foreign language number labels without reference either to specific objects in the laboratory or to Alex’s previously acquired English number labels [11,35]. The set, *il ee bam ba oo yuk chil gal*, was derived from Korean count labels both to permit comparisons with children [36] and to be maximally different from English numbers he already knew. (That is, his number labels used to distinguish actual quantities (i.e., cardinal numbers), “one”, “two”, etc. were to be kept distinct from his number labels used to distinguish position in a series (i.e., ordinal numbers), in a system not identical with, but related to, humans’ use of “first”, “second”, etc.). *Bam* (pronounced /baem/) and *ba* were substituted for the Korean *sam* and *sa* because of Alex’s occasional difficulty in producing “ss”. Alex thus did not initially observe any modeled connection between labels and their referents: that is, his input would lack as much reference and functionality as possible compared with his standard training [2,35]. Correct replies would receive vocal praise and the opportunity to request anything he desired (i.e., he would receive *x* for stating “I want x” [31], but *x* would have no referential relation to the count labels on which he was being trained); errors in the string (omissions, interchanging the order of elements) would elicit scolding, time-outs, and corrective feedback [35].

The training failed before we could even begin to collect data [35]. Although the plan was to have us train Alex in M/R variant 1 without any reference whatsoever, he would not attend (e.g., would turn his back and preen) until we included a minimal point of reference. We decided to use a sheet of paper with the symbols 1–8 printed along the diagonal. (NB: at that time, he did not know that his English number labels corresponded to these symbols; such training came later). In a typical session, a trainer showed the paper to the model and stated, “Say number?” (In contrast, queries in all previous number-related studies had been “How many?”). If the model correctly uttered the altered Korean labels in order, s/he was allowed to request a toy or food; errors resulting in mild scolding (e.g., “No, you’re wrong!”) and corrective feedback. As in basic M/R sessions, we routinely reversed roles of model and trainer; Alex was also given a turn. Alex’s only reward was the opportunity to request a favored item (“I want *x*”) for a correct response. Training, therefore, lacked functional meaning and all but minimal reference. The procedure did, however, maintain joint attention among bird, humans, and the pictured numbers.

Alex eventually did acquire the modeled string of number labels, but his results differed in two striking ways from those for utterances learned in standard M/R sessions [2,35]. First, whereas with standard input he would generally attempt new labels in just a few days of training and rarely needed more than a few weeks to achieve a recognizable reproduction, here he needed 9 months of training to learn the elements of the string. Second, and most interesting, was that he could not immediately use, nor subsequently learn to use, these labels referentially in any manner. He could merely repeat the labels by rote. Whether or not the paper with the printed numerals was present, he responded with the string whenever he heard “Say number?” Even after we modeled a correspondence of the string with various sets of items, he could not learn any 1:1 relationship between a set of eight objects and the string of labels, nor could he use elements in the string to refer to smaller quantities, e.g., to say “il ee bam” when presented with three items and queried “Say number?” Alex had thus learned to reproduce—that is, mimic—but not comprehend these human vocalizations [35]. I doubted that the task was too difficult, given his previous success on both production and comprehension of human labels after M/R training (e.g., [37,38]) and his ability to refer to sets of objects with his prior number labels [11]. I believed that his failure was, as predicted by social modeling theory, a consequence of the type of training—one lacking reference and functionality—and not a lack of general cognitive capacity [11].

### 5.3. M/R Variant 2

This variant examined what would happen if functionality was almost (but not quite) removed and social interaction was strongly reduced but not eliminated during training [39]. Based on studies with children [29,40], the point was specifically to examine the role of joint focusing of attention between bird and trainer on the object whose label was to be learned. Here, a single trainer faced away from a bird, who was within reach of the targeted object—for example, a key—that was suspended by a string, in view of the bird. The trainer talked about the object, emphasizing its label, “Look, a shiny *key*!” “Do you want *key*?”, etc. These sentences carefully framed the label, allowing for repeated label use while minimizing possible habituation [2,41]. The trainer had no visual or physical contact with the parrot or object but responded to the bird’s vocal behavior. Specifically, if a bird attempted to utter the targeted label, it would receive vocal praise (“That’s right, it’s a *key*!”), but never the object, although it could see the object to which the label referred. Two birds, Alo and Kyaaro, received such training on two labels in this variant, which was compared with their training on two other labels via the standard M/R protocol. Labels for this and all subsequent experiments were chosen according to the same criteria used for the audiotape study. We attempted to provide equivalent exposure to the several labels being trained (see [39]). Both birds learned the labels trained with the standard M/R protocol (and learned fairly quickly) but failed to learn the labels trained with the variant. The comparison showed that the training provided in this variant, not the birds’ basic abilities, affected their acquisition.

### 5.4. Basic Videotape Input

In “basic video” sessions, Kyaaro and Alo received input that demonstrated reference but avoided social interaction and minimized functionality in different ways than did M/R variant 2; again, labels trained in this manner were contrasted with other labels trained with the standard M/R procedure. Here, we again made videotapes of M/R sessions with Alex and two humans, then exposed the juveniles to those tapes [35], but now with the video portion intact. A zoom lens enabled us to include life-size images of Alex and the targeted objects in addition to the somewhat smaller images of the entire training scenario (the object, Alex, and two humans). Tapes also retained normal patterns of breaks for nonvocal exchanges (e.g., when trainers preened Alex) and trainers’ departures by using, respectively, scenes of such nonvocal interactions or a blank screen. The audio portion of the video was again analyzed to ensure that it was not degraded compared to that of Alex “live”. During sessions, each juvenile parrot was placed on a perch in a separate room so that the videos were viewed in isolation; thus, no direct social interaction with any humans occurred, although the birds saw the humans interacting with Alex and jointly attending to the object to be labeled. By watching a tape of a human or Alex produce a particular sound and either receive an object or be scolded, the juveniles saw but did not directly experience the effect of a vocalization. Videos, therefore, demonstrated reference but lacked all physical social interaction and clear functionality for the subject, who was now simply watching the video and could not receive any reward. (NB: our use of a cathode ray tube (CRT) screen—standard at the time—might have made learning from video impossible because of flicker fusion effects (e.g., [42]), distance, size of image, or lack of UV input [43]. Subsequent studies described below [44,45] take some of these issues into account). Again, birds learned the labels that were trained in M/R sessions, but not those trained via this video technique.

### 5.5. Video Variant 1

Because of research that (at least at the time I was performing these experiments) suggested that normal children learn more about labels from video instruction if they watch with an interactive adult who emphasizes important points, asks the children questions about what they are viewing, responds to the children’s questions, and even corrects the children’s comments (e.g., [46], but see [47] for children with language impairments), we decided to repeat the video study, this time providing the parrots with a trainer who would provide at least some interaction during viewing [48]. In our study, however, the trainer merely ensured—as described below—that the birds attended to the input and provided no physical referential reward if the bird identified something onscreen. That is, a bird that attempted the label received vocal praise but not the object. Trainers would provide social approbation for viewing and point to the screen, making comments (“Look what Alex has!”), but would not repeat new labels, ask questions, or relate content to other sessions [11]. Thus, the birds were not provided any real assistance in extracting information from the video. The amount of social interaction was limited and amount of functional meaning was the same as in basic video sessions. As before, labels trained in this manner were contrasted with labels trained via the M/R protocol; Kyaaro and Alo failed to learn labels introduced in this manner, although here they actually did make a few attempts at producing the label. Lack of the object as a reward, however, extinguished this behavior [48], and the majority of their vocalizations were not label attempts but rather utterances used to attract the attention of the trainer (e.g., “hello”) or labels for other objects that they had previously acquired during M/R sessions. As before, they learned the labels taught via the M/R protocol.

### 5.6. Video Variant 2

So far, all these experiments had eliminated most of the functionality from input—that is, the concrete reason for learning new labels as requests for desired items. To see what would happen in the absence of social interaction but with the possibility of functionality, in another experiment using videotaped input, we now added a system that could reward a parrot for attempting the label [44]. The system was controlled by a trainer in another room who monitored the parrot’s utterances through headphones. If the bird attempted the label, the trainer activated a pulley, lowering the object from a container to a spot within the bird’s reach. We audiotaped sessions to test for interobserver reliability of the parrots’ utterances during training. Of course, the bird had to make an attempt in order to be rewarded, and neither Kyaaro nor Alo did so. Again, no new labels were acquired, possibly because they had previously learned that attempts were not useful. From the birds’ point of view this experiment did not differ from the basic video, and by ignoring even their general squawks in that study, we may have extinguished their attempts at vocal behavior in the absence of a human. In retrospect, we could have modeled one session where a trainer received a reward for making an attempt, so that the birds would have understood the possibility of receiving an object in this situation via the pulley system. Thus, a variant of this experiment remains a future possibility.

### 5.7. Video Variants 3 and 4

Here we decided to examine the possibility that additional, though not full, social interaction—that is, a single interactive human—plus functionality and reference might, when added to video, engender learning [44], as it had to some extent with children [46,47]. Here the human co-viewer was more interactive, uttering targeted labels (but only in response to the actions on the video), querying Kyaaro and now Griffin about the video objects, and providing the object if the birds attempted the label, but live modeling was absent. Then, testing the assumption that the parrots might have failed to learn from video simply because they lost interest after seeing the same video repeatedly, we repeated the same amount of interaction and functionality for Griffin, but now with a live feed from sessions with Alex [44], so that the video input varied from session to session. Again, birds in these sessions experienced training on one set of labels in the video conditions and training with different labels in standard M/R sessions. Although Kyaaro did produce a few attempts at a targeted label during his recorded video sessions, he failed to do so either frequently or clearly enough for any acquisition to be noted; thus, he effectively learned nothing from his video sessions but, again, learned from M/R sessions [44]. Griffin behaved similarly in the recorded video sessions, although he made more attempts than did Kyaaro and interacted with the object more often; nevertheless, his vocalizations at the end of training were not distinctive enough to be tested. During live-feed video sessions, he made more attempts on one word (“chalk”) than he had during recorded sessions, but fewer for another (“bear”). The study was contaminated, however, because Alex (probably because of the additional exposure during live feeds) began to use “chalk” more frequently around trainers and in Griffin’s presence. We therefore did test Griffin on these labels as well as those acquired via M/R training; he failed to use the appropriate labels to identify the former objects but succeeded with the latter.

### 5.8. LCD Video Training

As noted above, our use of a CRT screen for all of our previous video work might have made learning difficult, if not impossible, because the screen did not reproduce UV (i.e., objects did not look the same as in real-life to parrots, who have UV vision [43]) and because parrots’ flicker-fusion rate would make the video seem like a flashing series of still photos. We were not overly concerned about the UV issue, because an earlier study [49] found that parrots, like humans, respond to videos of their toys despite possible color distortion. To test whether CRT screen flicker could have prevented learning, we ran a study with Griffin and Arthur using a liquid crystal display (LCD) monitor, which operates at refresh rate high enough (~200 Hz) to exceed the parrots’ flicker rate [50]. We replicated the protocol of video variant 1, because we were not attempting to determine optimal conditions for learning with video, but rather were determining if flicker-fusion was the critical issue. Both Arthur and Griffin produced numerous labels previously acquired during M/R training during the LCD video presentations, but almost no attempts at the actual labels being targeted [45]. Thus, although flicker-fusion might contribute to the lack of learning during video presentation, its elimination did not enable birds to learn from video under conditions of limited social interaction.

### 5.9. Single Trainer Input

In yet another attempt to determine what was critical for referential label acquisition in Grey parrots, an experiment with Griffin eliminated the model. Here, we contrasted standard M/R sessions with those in which a trainer labeled and handled objects to which she and Griffin jointly attended [51]. In Griffin’s presence, the human uttered phrases involving a targeted label (e.g., “Look at *x*!” or “I have an *x*!”), concurrently manipulating the corresponding item. Griffin thus saw behavior somewhat related to that in M/R sessions, where a trainer would say, “Yes, *you* have an *x*!” when rewarding the model/rival. The solo trainer demonstrated the association between label and item but did not model functional label use or errors. As in M/R sessions, Griffin was also queried about the object (“What’s here?”). Any of his attempts at the label would be noted and rewarded with the item; thus, only if he attempted a label would he experience its functionality. As in M/R training, if he erred on a label, a trainer would tell him to “Try again,” “Say better,” etc. We ensured that he was queried at the same rate and in the same way as in M/R training, so these solos differed only in lack of human-human modeling and were not a series of drills. Interestingly, Griffin did not utter any targeted label during or immediately after 50 sessions but did so after two or three subsequent M/R sessions involving the same items. Because birds usually need about 20 M/R sessions to fully and clearly produce a novel label (slightly faster if that label has significant phonetic overlap with an existent label [11]), Griffin had likely acquired labels during the solo-trainer sessions, but did not understand how to *use* them until he observed their usage modeled [51]. Such behavior differed from that in standard M/R sessions, where he learned new labels and their use concurrently, which he again did with the labels taught using the M/R protocol in this experiment.

### 5.10. M/R with a Minimally Interactive Conspecific

Here we wanted to see what might happen if a parrot experienced full reference and functionality, modeling, corrective feedback, but no role reversal, and if lack of role reversal might be mitigated by the use of a conspecific [51]. Thus, Griffin was trained with Alex and one human in an approximation of the standard M/R system. Alex could act as a model, and because he occasionally erred, could be corrected; however, at this stage he could not query Griffin. We note that he sometimes had done so informally, by interrupting Griffin’s M/R sessions (e.g., if we were asking Griffin about the color of an item and Griffin had mumbled his response, Alex might insert “Say better!”), but he did not actually pose specific questions at that time. (Note that Alex did understand the use of questions, and asked *trainers* for labels for novel items, colors, shapes, etc.; maybe he understood who was an appropriate source of information, and that Griffin was not? Only several years later were we able to train Alex to query Griffin). Unlike most of the other modifications to the M/R procedure, this one had some success. Griffin learned two labels trained in these sessions. Acquisition was, however, notably and considerably slower than in the standard M/R procedure, requiring about twice as many sessions for acquisition to occur. Many possible reasons exist for this slower rate of acquisition aside from the lack of role reversal; these are discussed in detail in [51]. Another possibility, however, was that Griffin saw the interactions as a form of duetting and bonding between the human and Alex—who was definitely the dominant parrot in the lab—and maybe Griffin, as the subordinate, feared to interrupt.

## 6. Implications of the Experiments on M/R Training Variants

The numerous experiments described above clearly could not examine every combination of various possible levels of input with respect to reference, functionality, and social interaction. For example, we did not test what would happen if, in the standard M/R condition, models never erred and thus the birds never saw corrective feedback. (Given studies at the time with pigeons and starlings on other types of tasks, showing how observing models’ errors improved performance [52,53,54], we suggest that learning would have occurred after training without error demonstrations and feedback, but at a slower pace). However, our experiments overall do at least show the importance of specific types of input (Table 1): that two live models who demonstrate reference, functionality, joint attention, and extensive social interaction are necessary but not entirely sufficient for referential label acquisition [11,28]. Why necessary but not sufficient? We deduce this training is necessary because such learning did not occur through the use of (a) audio tapes in isolation; (b) a single trainer who acted referentially but did not demonstrate functionality; (c) a single trainer who also did not interact with the bird; (d) videotapes featuring two trainers (and a parrot) who exhibited the same behavior as live M/R models, including role reversal and corrective feedback of errors; (e) two live models with reversal with no functionality and extremely limited reference; or (f) a solo trainer plus the same videotaped material [35,39,44,48]. Todt’s training [8], which lacked explicit referentiality, role reversal, and error demonstration, engendered facile learning, but without generalization to individuals or situations beyond the training situation. Our data also show that in the absence of two live models, role reversal and error demonstration are also insufficient [35]. We deduced that live two-trainer modeling, including functionality, referentiality, and demonstration of corrective feedback of a model’s errors but without role reversal, engendered learning but at a far slower pace [51]. Thus, the many elements of input are each necessary, but only the presence of *all* together provides sufficient means for full and rapid acquisition to occur; lack of any one of them may result in various levels of failure.

## 7. Why Do We Need Interspecies Communication?

I have argued previously (summarized in [4,5,55,56]) that training on symbolic representation, and the form of interspecies communication it engenders, affects the ease with which animals learn and can be tested on certain concepts. Furthermore, as did Premack before me [3], I suggest that a system of two-way communication may enable a researcher to teach a concept that an animal subject may not easily acquire by other means, and, most importantly, that acquisition of symbolic representation can even affect how subjects manipulate information.

Premack [3] presented evidence for chimpanzees, and I for Grey parrots [4,56], showing that symbolic reference enables (but, of course, cannot guarantee) abstract thought. Thus, once a nonhuman understands that a symbol can be used to represent an object, an action, an attribute, etc., the subject can then mentally manipulate that symbol. Such mental manipulation releases thought processes to, for example, enable formation of concepts, insight, and transfer to novel situations. A subject can plan, then mentally—rather than physically—test the consequences of choices. I have previously described, in detail, the several studies on Grey parrots in which I believe symbolic representation and interspecies communication have facilitated learning (e.g., [4,5,56]) and, thus, will only summarize this material here.

### 7.1. Studies Facilitated by Interspecies Communication

In some studies, symbolic processing plays an important role not specifically because it enables more complex processing, but because it facilitates training and/or eliminates confounds that can detract from the claims of success in other species. In each of these studies, the parrot could be taught a label for a concept, rather than being required to derive the concept over thousands of trial-and-error tests, and then could be exposed to transfer trials to examine how well the concept was understood.

For example, in studies of optical illusions, humans are simply asked what they see. However, such is rarely the case for nonhumans who are tested to determine how their perception of the same stimuli compares to that of humans. Nonhumans instead are generally subjected to intensive training procedures to enable them to discriminate the initial stimulus, followed by tests on their recognition of similar patterns; results often are contingent upon statistical averaging over hundreds of trials of pecking/touching behavior to a very limited set of choices. The data thus are often highly variable and dependent upon details of the experimental design (reviewed in [57]). Alex, in contrast, could simply be shown pictures of the Müller–Lyer illusion (Brentano version), in which the two horizontal lines were of different colors, and, as he had previously acquired the concept of relative size [58], be asked, “What color bigger/smaller?” [57]. Likewise, once another Grey parrot, Griffin, had learned labels for shapes, and thus that a vocal label could represent an object, he could understand how two symbols (e.g., one vocal and one visual), which separately represent the same object, can then represent each other (a form of equivalence [59]) and thus how, for example, a three-dimensional entity can be represented by a two-dimensional drawing, so that he could appropriately identify pictures of occluded objects and Kanizsa figures (i.e., other optical illusions) [60]. (Possibly, one might also therefore argue that this study was critically dependent upon symbolic representation; see below).

It is also likely that Griffin’s understanding of symbolic representation assisted him in other experiments. That is, he might have used symbols as place-markers to assist in tasks requiring memory [61,62] and evaluation of probability [63]. Even limited understanding of symbolic representation may have helped other Grey parrots in tasks such as Piagetian liquid conservation [64] and inference by exclusion [65,66], where memory for specific quantities or objects, respectively, is also of considerable importance.

### 7.2. Studies Critically Dependent upon Symbolic Representation

As noted above, Premack [3] has argued that acquisition of symbolic representation not only enables nonhumans to be tested more easily, but also may actually enable them to perform tasks that are failed by nonhumans lacking such representation. Some of the tasks I describe below I had previously listed in the former category (e.g., see [56]), but now I believe they may fall into the latter. Here, I believe that symbolic representation has been a crucial tool for studying the extent of Grey parrot cognitive capacity.

For example, most nonhumans have considerable difficulty demonstrating an understanding of relational concepts, such as ‘bigger–smaller’, unlike tasks involving concrete concepts such as color, where something that is “red” is always “red”, in relational tasks the sample that is, for example, the smaller object on one trial (e.g., a tennis ball versus a basketball) can be the bigger object on another (e.g., the tennis ball versus a golf ball). Most subjects are trained to derive one concept over large numbers of trials (i.e., by being rewarded for choosing only the bigger item) and then the other through large numbers of reversals (i.e., now being rewarded for choosing only the smaller)—in this training paradigm, however, both concepts may actually never be acquired, in that a subject without symbolic reference may simply learn “choose X” versus “avoid X” (see [67,68] for a discussion). In contrast, my Grey parrot, Alex, who learned to respond to “What color bigger/smaller?” for three sets of items, was then able to transfer his responses, without additional training, to a large number of sets involving sizes outside the training paradigm and to totally novel objects with respect to shape, color, and material [58]; he also spontaneously transferred his understanding of the concept to questions for which the responses involved material rather than color, and spontaneously transferred use of the label “none” (acquired in a different study, [69]) to respond appropriately when the two objects were of the same size [58].

The ability to comprehend concepts of same-different is the specific one that Premack [3] used to buttress his argument for the effect of symbolic representation on nonhuman cognitive processing. Details of that argument have been the subject of an entirely separate paper [4], but the point is that nonhumans lacking symbolic representation do not fully comprehend same–different, whereas those with symbolic representation do. The issue is that same–different is not merely recognition of identity versus non-identity (i.e., whether two stimuli are completely equal or completely unequal in every possible aspect) or a difference in entropy (i.e., in overall randomness) between stimuli sets (e.g., [70]). Rather, it is a task that, according to Premack’s stringent criteria [3], requires a feature analysis of the objects being compared, recognition that objects can simultaneously exhibit attributes that involve both similarity and difference, and the ability to understand which attributes are being targeted based on questions of either similarity or difference. As noted in [6,7], an appropriate response requires a subject to (a) attend to multiple aspects of two different objects; (b) determine, from a verbal question, whether the response is to be based on sameness or difference; (c) determine, from the exemplars, exactly what is same or different (i.e., what are their colors/shapes/materials?); and then (d) produce, verbally, the label for the hierarchical category of the appropriate attribute (i.e., “color”, “shape”, “matter”). The task is therefore a clear instance in which symbolic reference is critical for success. Not only did the Grey parrot, Alex, succeed [4,71] but he transferred his knowledge to a completely novel instantiation of the same-different concept (i.e., something beyond his familiar comparisons of color/shape/material) without additional training. After he learned to use the symbols “same” and “different” to represent the relations amongst specific categories for object pairs, he spontaneously understood how the same–different relationship applied to an abstract representation of a novel situation—as when, queried for the first time “What color bigger?” for two equally-sized items, he asked “What’s same?” and then responded “none” (see above, [58]). Such fluid response ability requires symbolic, referential, interspecies communication.

Another topic in which symbolic representation plays a major role is number concepts. Lacking symbols for specific quantities, individuals may still demonstrate some sense of number. Using what is termed the Approximate Number System (ANS), almost every nonhuman tested plus pre-verbal children can distinguish exact quantities for sets up to about three [72]. The ANS, however, cannot identify quantities precisely for amounts much above that number; to do so, individuals need actual symbols [73]. Such understanding of numerical symbols was once thought to be limited to humans, but several nonhumans—two apes and the Grey parrot, Alex—learned to use Arabic numerals to distinguish quantities up to eight or nine (reviewed in [34]). These subjects could produce the correct numerical label in the presence of specific sets, could identify the correct set in the presence of the corresponding Arabic numeral, understood how to add small quantities [33,74], could learn [75] or infer [32] the ordinality of the numerals after demonstrating an understanding of their cardinality, and all had a zero-like concept (reviewed in [34]). The parrot could also infer the cardinality of a novel number after learning its ordinal value in a number line [34]. Such abilities can be approached by the ANS, but are not fully possible without exact symbolic reference [73].

## 8. Discussion and Conclusions

Only a very small number of individual nonhumans have acquired the form of symbolic representation described here—a few nonhuman primates, a few marine mammals, a few Grey parrots (summarized in [76])—and such studies are, for the most part, no longer in progress [76]. Some dogs have acquired some labels as well (note [77]). The primary point of this paper was not to review the overall field of interspecies communication, but rather to discuss the processes involved in the acquisition of such communication, specifically from the standpoint of a set of studies on Grey parrots. I suggest that the reason that so few experiments in the field have been successful probably lies less in the capacities of the various nonhumans that were trained, but rather more with the capacities of the humans performing the training. Such training must be done with extreme attention to detail so that all the elements of input that are both necessary and sufficient are present, otherwise learning will either not occur or be incomplete: the full extent of any nonhuman capacity for symbolic representation will not be achieved.

It is important to recognize that none of the nonhuman individuals in these studies on symbolic representation acquired all the complexities that are inherent in human language (even with what one hopes was fully appropriate training); much is yet to be learned about the reasons why. Nevertheless, the acquired level of interspecies communication in this unique subset did enable an examination of their cognitive capacities in ways that would have been much more difficult, if not impossible, by other means. Again, I have not tried to review the entire field of animal cognition, but have focused on my specific area of expertise, the abilities of Grey parrots. Given that these birds are evolutionarily separated from mammals by over 320 million years of evolution [78], and yet have demonstrated abilities in many ways comparable to those of 5–8 year old children (e.g., [4,5,32,33,34,36,37,38,39,62,63,64,65,66], one wonders what other individuals of various species might also demonstrate advanced cognition if tested in an appropriate manner?

## Figures and Tables

**Table 1:** Components and results of various types of tutoring regimes.

Regime	Reference	Functionality	Social Interaction	Parrot	Learning?
M/R	Yes	Yes	Full model	All	Yes
Audiotape	No	No	No	Alo, Kyaaro	No
M/R variant 1	No	No	Full model	Alex	Mimicry
M/R variant 2	Yes	Minimal	Minimal	Alo, Kyaaro	No
Basic video	Yes	Partial	No	Alo, Kyaaro	No
Video variant 1	Yes	Partial	Minimal	Alo, Kyaaro	Minimal
Video variant 2	Yes	Potential	No	Alo, Kyaaro	No
Video variants 3, 4	Yes	Potential	Some	Kyaaro, Griffin	No
LCD video	Yes	Partial	No	Kyaaro, Griffin	No
Single trainer	Yes	Potential	Yes, no model	Griffin	Hidden
Conspecific	Yes	Yes	Yes, no reversal	Griffin	Slow
